# Supplementary material for: Understanding the role of veterinarians in antimicrobial stewardship on Canadian dairy farms: A mixed-methods study
Source: PLoS One. 2023 Jul 27;18(7):e0289415. doi: 10.1371/journal.pone.0289415 (PMC10374071; doi:10.1371/journal.pone.0289415)
Supplement: S1 Appendix — (DOCX) [file pone.0289415.s001.docx]

Understanding the role of veterinarians in antimicrobial stewardship in dairy farms

Start of Block: Block 1

University of Guelph
 Consent to Participate in Research

**Study title: Understanding the role of veterinarians in antimicrobial stewardship on Canadian dairy farms**

 We invite you to participate in a study aiming to explore veterinarian’s drivers and barriers for improving antimicrobial stewardship on dairy farms in Canada. Our results will inform future programs in Canada to reduce the use of antimicrobials in dairy farming that will help to combat the antimicrobial resistance problem and keep antimicrobials working for all of us.
This research is funded by the Food From Thought Research Program of the University of Guelph and is conducted by Dr. Stephen LeBlanc (principal investigator) and Dr. Claudia Cobo Angel. Participation is voluntary. Your decision to participate or not will not have repercussions in any way. You do not waive any legal rights by agreeing to take part in this study. Your participation in this study will not affect your participation in other studies in which you are already enrolled, or you might be planning to enroll.
 If you agree to participate in this study, you will respond to questions related to your considerations for prescribing antimicrobials, and your attitudes and awareness toward antimicrobial use and antimicrobial resistance. In addition, we will collect some demographic and identifiable information such as your name and email. To answer all questions will take you about 15 minutes, and you will be able to skip any questions that you do not want to answer. Furthermore, you can quit the questionnaire or withdraw from the study without repercussions. You have up to two weeks after completing the questionnaire to let us know about any of answers that you do not want us to use in analysis and publications.
We are using a secure system to collect this data, but any time you share information online there are risks. We are minimizing this risk that your information would be seen by others who should not have access to it by removing all identifying information and replacing it with a study ID. We will keep your identifying information separate from your research data, but we will be able to link it to you. After analysis and publication of the results, we will destroy the link between the research data and any identifying information, unless you agree to be contacted for further studies. The results of this study will be published in academic journals and presented at industry and academic conferences; however, we will not use information that will enable identification of participants in any way. You will have the option to receive a summary of the findings after the study. 
 If you have any questions or concerns about the study, please contact Claudia Cobo at ccobo@uoguelph.ca. Please print or save this screen if you want to be able to access the information later.
 This project has been reviewed by the Research Ethics Board for compliance with federal guidelines for research involving human participants. If you have questions regarding your rights and welfare as a research participant in this study (REB #21-10-025), please contact Research Ethics; University of Guelph (reb@uoguelph.ca; 519824-4120 ext. 56606).

| 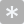 |
| --- |

If you want to participate, please click to confirm that:

- You are at least 18 years old (1)
- You understand that you can withdraw at any time and/or not answer any question (2)
- You understand that you have two weeks after completing the questionnaire to let us know about any of your answers or data that you do not want us to use in publications (3)
- You consent to participate in this study (4)

End of Block: Block 1

Start of Block: General information

Please indicate the gender pronoun with which you identify

- He/Him (1)
- She/Her (2)
- They/Them (3)
- I prefer not to say (4)

Please select your province or territory of residence

- Alberta (1)
- British Columbia (5)
- Manitoba (6)
- New Brunswick (7)
- Newfoundland and Labrador (8)
- Northwest Territories (9)
- Nova Scotia (10)
- Nunavut (11)
- Ontario (12)
- Prince Edward Island (13)
- Quebec (14)
- Saskatchewan (15)
- Yukon (16)

Please indicate your age

________________________________________________________________

How many years of experience working with dairy cattle as a licensed veterinarian do you have?

________________________________________________________________

Please indicate your current position (please select the most applicable)

- Associate in a private veterinary clinic (1)
- Owner or partner in a private veterinary clinic (2)
- Working for an academic institution (3)
- Other (4)

Please specify

________________________________________________________________

| Page Break |  |
| --- | --- |

End of Block: General information

Start of Block: Block 3

Thinking of your last 10 farm visits to dairy farms, how many times did you prescribe or provide antimicrobials?

- None (1)
- 1 to 3 (2)
- 4 to 6 (3)
- 7 to 9 (4)
- All 10 (5)

Once you have made the decision to prescribe an antibiotic, how important to you are the following reasons to select a particular antibiotic product?

|  | Not at all important (1) | Slightly important (2) | Moderately important (3) | Very important (4) | Extremely important (5) |
| --- | --- | --- | --- | --- | --- |
| Previous experience with the antimicrobial (1) |  |  |  |  |  |
| Knowledge about the antimicrobial (2) |  |  |  |  |  |
| Colleagues’ reports of efficacy (3) |  |  |  |  |  |
| Clients’ reports of efficacy (4) |  |  |  |  |  |
| Peer-reviewed papers on efficacy (5) |  |  |  |  |  |
| Marketing materials (6) |  |  |  |  |  |
| Technical information from manufacturers (7) |  |  |  |  |  |
| Clinic policy (8) |  |  |  |  |  |
| Client’s request (9) |  |  |  |  |  |
| Client’s quota situation (whether they are filling quota or not) (10) |  |  |  |  |  |

| Page Break |  |
| --- | --- |

How important to you are the following factors **related to the antimicrobial compound** (as opposed to the brand of the product) when selecting a particular product

|  | Not at all important (1) | Slightly important (2) | Moderately important (3) | Very important (4) | Extremely important (5) |
| --- | --- | --- | --- | --- | --- |
| Withholding period (1) |  |  |  |  |  |
| Spectrum of efficacy (2) |  |  |  |  |  |
| Duration of effect (3) |  |  |  |  |  |
| Route of administration (4) |  |  |  |  |  |
| Number of doses required (5) |  |  |  |  |  |
| On-label indication (6) |  |  |  |  |  |
| Own experience of efficacy (7) |  |  |  |  |  |
| Category of importance to human medicine (8) |  |  |  |  |  |
| Microbiological culture and susceptibility test (9) |  |  |  |  |  |

What other factors (related to the antimicrobial compound) do you consider when selecting a particular product?

________________________________________________________________

| 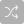 |
| --- |

How important to you are the following factors **related to the animal** that you consider when selecting a particular antimicrobial product?

|  | Not at all important (1) | Slightly important (2) | Moderately important (3) | Very important (4) | Extremely important (5) |
| --- | --- | --- | --- | --- | --- |
| Diagnosis (i.e., the condition being treated) (1) |  |  |  |  |  |
| Level of milk production (2) |  |  |  |  |  |
| Stage of lactation (3) |  |  |  |  |  |
| Weight (4) |  |  |  |  |  |
| Value of animal (5) |  |  |  |  |  |
| Animal safety (6) |  |  |  |  |  |
| Previous treatments (7) |  |  |  |  |  |

What other factors (related to the animal) do you consider when selecting a particular product?

________________________________________________________________

| Page Break |  |
| --- | --- |

| 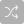 |
| --- |

How important to you are the following factors **related to the farm** that you consider when selecting a particular antimicrobial product?

|  | Not at all important (1) | Slightly important (2) | Moderately important (3) | Very important (4) | Extremely important (5) |
| --- | --- | --- | --- | --- | --- |
| Herd size (1) |  |  |  |  |  |
| Disease history of the farm (2) |  |  |  |  |  |
| History of on-farm response (3) |  |  |  |  |  |
| Housing type (4) |  |  |  |  |  |
| Milk quota situation (5) |  |  |  |  |  |
| Farmer's goals and preferences (6) |  |  |  |  |  |
| Expected compliance with the treatment prescribed (7) |  |  |  |  |  |
| Milking frequency (8) |  |  |  |  |  |

What other factors (related to the farm) do you consider when selecting a particular product?

________________________________________________________________

| Page Break |  |
| --- | --- |

Thinking of the last 12 months, how often have you performed a microbiological culture?

- Frequently (>75% of the time I deal with an infectious disease) (1)
- Often (50% to 75% of the time that I deal with an infectious disease) (2)
- Sometimes (5 to 50% of the time that I deal with an infectious disease) (3)
- Rarely ( (5)
- Never (6)

Thinking of the last 10 times you have performed a microbiological culture, how many times have you performed a susceptibility test?

- All 10 times (1)
- 6-9 times (2)
- 1-5 times (3)
- None (4)

| 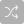 |
| --- |

What factors influence your decision to perform a microbiological culture and antibiotic susceptibility test? Rank in order of importance (click and drag):

______ Cost of the test (1)

______ Disease history of the farm (2)

______ Disease history of the cow (3)

______ Treatment failure (i.e., lack of improvement after treatment) (4)

______ Recurring infection (i.e., recurrence after apparent cure) (5)

What other factors do you consider when deciding to perform a microbiological culture and antibiotic susceptibility test??

________________________________________________________________

When do you usually recommend extended antimicrobial treatment (i.e., for longer than the duration on the label)? Select all that apply

- Never (1)
- When I deal with persistent infections (i.e. not resolved after the initial course of treatment) (2)
- When I deal with recurring infections (I.e., resolved after the initial course of treatment, but then returned) (3)
- For the initial course of treatment based on my experience (4)
- For the initial course of treatment based on my knowledge or reference materials (5)

End of Block: Block 3

Start of Block: Antimicrobial use and antimicrobial resistance awareness

Please indicate your level of agreement with the following statements about antibiotic use and antibiotic resistance on dairy farms:

|  | Strongly disagree (1) | Somewhat disagree (2) | Neither agree nor disagree (3) | Somewhat agree (4) | Strongly agree (5) |
| --- | --- | --- | --- | --- | --- |
| There is overuse of antimicrobials in dairy production (1) |  |  |  |  |  |
| We should reduce the use of antimicrobials in dairy production (2) |  |  |  |  |  |
| I could explain what antimicrobial resistance is and its causes to my clients (3) |  |  |  |  |  |
| Antimicrobial resistant infections are an important problem in dairy cattle (4) |  |  |  |  |  |
| The use of antimicrobials on a farm could cause antimicrobial resistance on that farm (5) |  |  |  |  |  |
| The use of antimicrobials on one farm could cause antimicrobial resistance on other farms (6) |  |  |  |  |  |
| The use of antimicrobials on dairy farms could cause antimicrobial resistance in humans (7) |  |  |  |  |  |
| When I prescribe antimicrobials, I try to avoid critically important antimicrobials for human medicine (8) |  |  |  |  |  |
| When I prescribe an antimicrobial, I think about the risk of antimicrobial resistance in cattle (9) |  |  |  |  |  |
| When I prescribe an antimicrobial, I think about the risk of antimicrobial resistance in humans (10) |  |  |  |  |  |
| I have had a discussion with my clients about when and how to use antimicrobials for treatments when they don’t consult with me (11) |  |  |  |  |  |
| Antimicrobials with no milk withholding time are less likely to cause antimicrobial resistance than those that require a withdrawal period (12) |  |  |  |  |  |
| Antimicrobial resistant infections in people are an important problem (13) |  |  |  |  |  |
| Antimicrobial resistance is an issue that could affect me or my family (14) |  |  |  |  |  |
| Antimicrobial resistance is an issue in other countries but not in Canada (15) |  |  |  |  |  |
| Veterinarians are responsible for AMR in animals (16) |  |  |  |  |  |
| Veterinarians are responsible for AMR in humans (17) |  |  |  |  |  |
| Veterinarians generally prescribe responsibly (18) |  |  |  |  |  |
| Farmers have limited understanding of antimicrobial resistance (19) |  |  |  |  |  |

End of Block: Antimicrobial use and antimicrobial resistance awareness

Start of Block: Antimicrobial use reduction

Do you think that milk production will be reduced if antimicrobial use is decreased?

- Definitely not (1)
- Probably not (2)
- Might or might not (3)
- Probably yes (4)
- Definitely yes (5)

Do you think that animal welfare would be worse if antimicrobial use is decreased?

- Definitely not (1)
- Probably not (2)
- Might or might not (3)
- Probably yes (4)
- Definitely yes (5)

| Page Break |  |
| --- | --- |

16. Should there be more initiatives to promote responsible use of antimicrobials in the dairy industry?

- Yes (1)
- No (2)

If yes, select one action that you consider would be the most effective measure to reduce use

- Put more regulations in place to change or reduce use of antimicrobials (1)
- Provide more education or promotion to change or reduce use (2)
- Measure use and provide benchmarking to compare use among dairy farms (3)
- Measure use and provide benchmarking to compare use among dairy veterinarians (4)
- Provide incentives to change or reduce use (5)

In your opinion, in what group of animals would it be easiest to reduce antimicrobial use without causing harm?

- Pre-weaned calves (1)
- Weaned calves and pre-breeding heifers (2)
- Breeding age and pregnant heifers (3)
- Lactating cows (4)
- Dry cows (5)

Thinking of the last 100 antibiotic treatments (not including dry cow therapy) administered on your clients’ dairy farms, on what proportion of cases do you think you were you directly consulted (by attending the animal or by phone or text)?

- >80% (1)
- 60 to 80% (2)
- 40 to 60% (3)
- 20 to 40% (4)
- < 20% (5)

Thinking of the last 100 antibiotic treatments (not including dry cow therapy) administered on your clients’ dairy farms, on what proportion of cases do you think the client followed a treatment protocol that you had provided?

- >80% (1)
- 60 to 80% (2)
- 40 to 60% (3)
- 20 to 40% (4)
- < 20% (5)

May we contact you regarding participating in a future intervention study to improve the use of antibiotics in dairy cattle?

- Yes (1)
- No (2)

May we contact you regarding participating in a focus group of veterinarians on use of antibiotics in dairy cattle?

- Yes (1)
- No (2)

In case you would like to be contacted in further studies, please write your email:

________________________________________________________________

Do you have additional comments that you want to share with us?

________________________________________________________________

End of Block: Antimicrobial use reduction

Start of Block: Block 2

Please choose the option that applies to you:

- I would like to receive a summary of the results (1)
- I would like to be contacted for future studies (in this case your identifiable information will be retained by the principal investigator) (2)
- I would like to receive a copy of the consent: (3)

End of Block: Block 2
