# Supplementary material for: Understanding the role of veterinarians in antimicrobial stewardship on Canadian dairy farms: A mixed-methods study
Source: PLoS One. 2023 Jul 27;18(7):e0289415. doi: 10.1371/journal.pone.0289415 (PMC10374071; doi:10.1371/journal.pone.0289415)
Supplement: S2 Appendix — (DOCX) [file pone.0289415.s002.docx]

**Understanding the role of veterinarians in antimicrobial stewardship on dairy farms**

**Semi-structured interview guide**

General aim: to explore the drivers and barriers for improving antimicrobial stewardship on dairy farms and attitudes toward AMU reduction and AMR

1. **Introduction**

Claudia: Welcome and thank you for participating in this focus group. My name is Claudia Cobo, I’m a Post-doctoral researcher at the department of population medicine of the University of Guelph. My research aims to understand how we can improve antimicrobial stewardship on Canadian dairy farms. We conducted focus groups with farmers and now we want to hear your perspectives regarding antimicrobial use, antimicrobial resistance, and antimicrobial stewardship. This activity will be moderated by Steve and will take about an hour and a half. Once again thanks for being part of our research.

1. **Drivers for antimicrobial prescribing**

I would like to know more about your experience and how you make decisions regarding antimicrobial treatments.

Q1. Think about the last few times you prescribed or recommended antimicrobial treatment. What factors go into your decision to treat with an antimicrobial?

Probe if necessary: type of disease, age of the animal(s), type of antimicrobial, waiting period for starting the antimicrobial treatment

Q2. Think about a recent consultation in which you were in doubt about prescribing antimicrobials. Can you describe it?

What factors weighed for and against using an antimicrobial?

Q3. How do you usually select which antimicrobial to prescribe?

Probe: farm factors, animal factors, antimicrobial characteristics.

Probe: In what situations would you perform a bacteriologic culture and susceptibility test?

Q4. Is there a policy on AMU in your clinic? If yes, when do you stick to this policy and when don’t you?

Q5. In what situations would you recommend extra-label antimicrobial treatment? For example, extended treatment?

*External influences*

Q6. How do farmers expectations of how they think the animal should be treated influence your decision-making around antimicrobial use?”

Q7. How does the cost of treatment influence decision-making?

Q7. How do your colleagues affect your antimicrobial prescribing decisions?

Probe: do you discuss antimicrobial treatment decisions with your colleagues?

Q8. How do pharmaceutical companies affect your antimicrobial prescribing decisions?

Q9. How does the current public discussion on AMU/AMS influence your decisions?

Probe: Are there other (not mentioned) parties/factors affecting your AM prescribing behavior?

1. **Awareness of AMR and attitudes toward AMU reductions**

As you know, there is global pressure to reduce the AMU in agriculture and specifically in food-producing animals, due to the development of antimicrobial resistant infections in humans.

Q10.What is your opinion on the public discussion on AMU in farm animals?

Q11. What do you consider as “responsible AMU”?

Q12. To what extent do you think that antimicrobial resistance is a problem?

Probe: in dairy cattle and in humans

Q13. Do you consider that dairy farming plays an important role in human resistant pathogens?

Q14. To what extent is there overuse of AM in dairy production?”

Q15. In your opinion, who is responsible for promoting strategies to use antimicrobials more prudently on dairy farms?

Probe: government, milk companies, veterinarians, academia

Q16. In your opinion, what kind of strategies/actions are needed to reduce the use of antimicrobials in dairy farms?

Q17. How do you think that AMU reduction would affect dairy cattle health, production, or welfare?

Q18. In your opinion, what are the main barriers to reduce the use of antimicrobials on Canadian dairy farms?

Q19. Do you have any other comments regarding antimicrobial stewardship drivers and barriers that we haven’t discussed?
