# Supplementary material for: Understanding the role of veterinarians in antimicrobial stewardship on Canadian dairy farms: A mixed-methods study
Source: PLoS One. 2023 Jul 27;18(7):e0289415. doi: 10.1371/journal.pone.0289415 (PMC10374071; doi:10.1371/journal.pone.0289415)
Supplement: S1 Table — (DOCX) [file pone.0289415.s003.docx]

S1 Table. Detailed demographic information of the 26 participants of the focus groups.

| Participant | Focus group | Pronoun | Age | Years of experience working with dairy cattle as a licensed veterinarian | Current position |
| --- | --- | --- | --- | --- | --- |
| 1 | 1 | She/Her | 43 | 17 | Owner or partner in a private veterinary clinic |
| 2 | 1 | He/Him | 33 | 5 | Owner or partner in a private veterinary clinic |
| 3 | 1 | He/Him | 67 | 41 | Associate in a private veterinary clinic |
| 4 | 1 | He/Him | 35 | 12 | Owner or partner in a private veterinary clinic |
| 5 | 1 | He/Him | 36 | 10 | Owner or partner in a private veterinary clinic |
| 6 | 1 | She/Her | 30 | 6 | Associate in a private veterinary clinic |
| 7 | 2 | He/Him | 67 | 43 | Associate in a private veterinary clinic |
| 8 | 2 | He/Him | 59 | 34 | Owner or partner in a private veterinary clinic |
| 9 | 2 | He/Him | 67 | 41 | Owner or partner in a private veterinary clinic |
| 10 | 2 | She/Her | 34 | 9 | Associate in a private veterinary clinic |
| 11 | 3 | He/Him | 34 | 7 | Associate in a private veterinary clinic |
| 12 | 3 | He/Him | 35 | 10 | Owner or partner in a private veterinary clinic |
| 13 | 3 | She/Her | 30 | 5 | Owner or partner in a private veterinary clinic |
| 14 | 3 | He/Him | 54 | 30 | Owner or partner in a private veterinary clinic |
| 15 | 3 | She/Her | 37 | 11 | Owner or partner in a private veterinary clinic |
| 16 | 3 | She/Her | 40 | 15 | Owner or partner in a private veterinary clinic |
| 17 | 3 | She/Her | 40 | 10 | Associate in a private veterinary clinic |
| 18 | 4 | She/Her | 25 | 1 | Associate in a private veterinary clinic |
| 19 | 4 | She/Her | 32 | 6 | Associate in a private veterinary clinic |
| 20 | 4 | He/Him | 41 | 15 | Owner or partner in a private veterinary clinic |
| 21 | 4 | She/Her | 29 | 1 | Working for an academic institution |
| 22 | 4 | He/Him | 69 | 39 | Owner or partner in a private veterinary clinic |
| 23 | 4 | He/Him | 49 | 21 | Owner or partner in a private veterinary clinic |
| 24 | 4 | She/Her | 28 | 5 | Associate in a private veterinary clinic |
| 25 | 4 | He/Him | 34 | 8 | Owner or partner in a private veterinary clinic |
| 26 | 4 | She/Her | 31 | 5 | Associate in a private veterinary clinic |
