# Supplementary material for: Understanding the role of veterinarians in antimicrobial stewardship on Canadian dairy farms: A mixed-methods study
Source: PLoS One. 2023 Jul 27;18(7):e0289415. doi: 10.1371/journal.pone.0289415 (PMC10374071; doi:10.1371/journal.pone.0289415)
Supplement: S1 File — (DOCX) [file pone.0289415.s004.docx]

**SUPPLEMENTARY FIGURES**

**Understanding the role of veterinarians in antimicrobial stewardship on Canadian dairy farms: a mixed-methods study**

Claudia Cobo-Angel*^1,2&¶^; Steven M. Roche^1,3&^; Stephen J. LeBlanc^1&¶^

^1^ Department of Population Medicine, University of Guelph, Guelph, ON, Canada.

^2^ Cornell University College of Veterinary Medicine, Ithaca, NY, United States.

^3^Agricultural Communications & Epidemiological Research (ACER) Consulting Limited, Guelph, ON, Canada


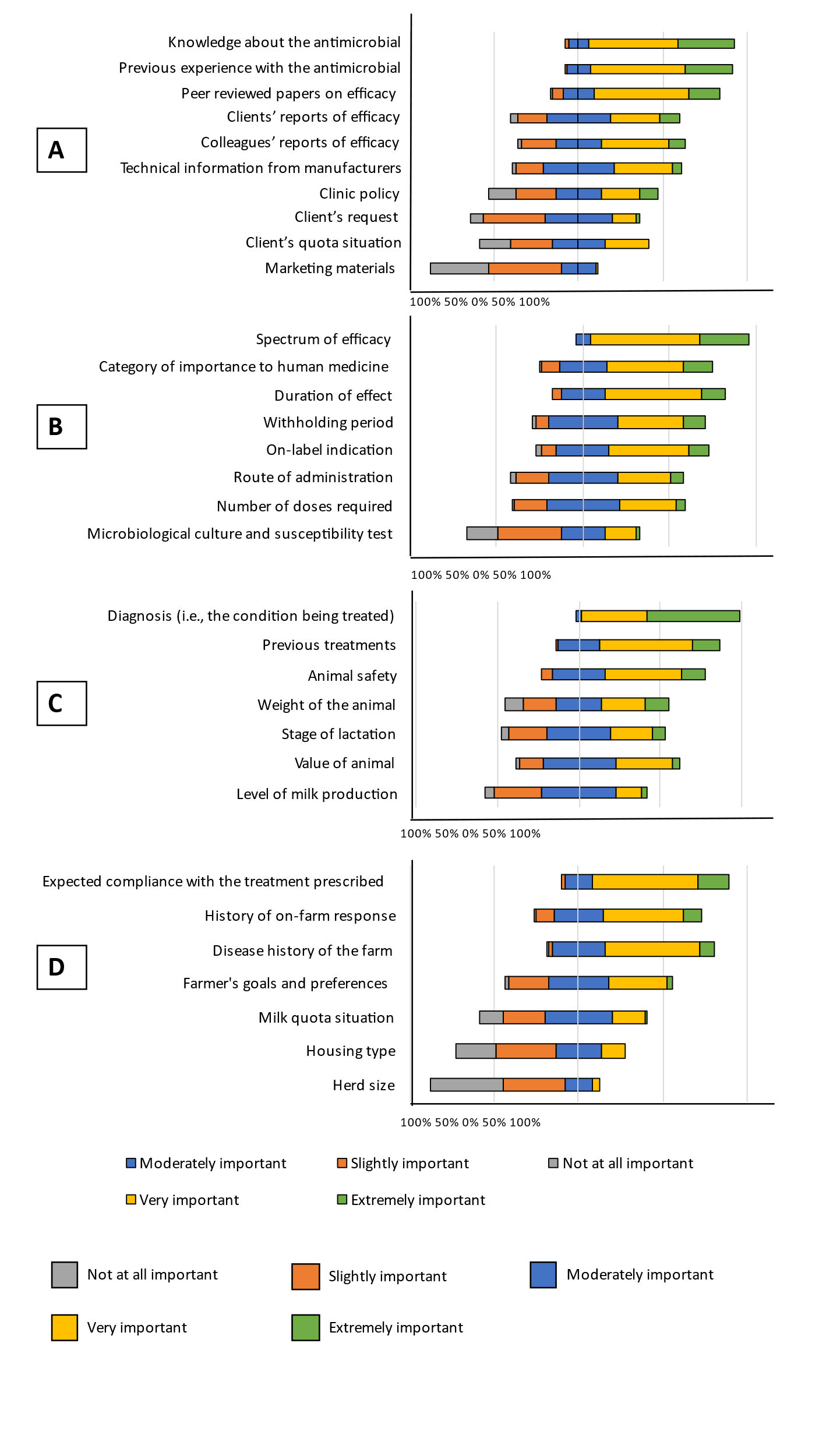


Supplementary Figure S1. The proportions of 81 responses to a survey of Canadian dairy cattle veterinarians regarding the level of importance of factors to select an antimicrobial treatment in general (A), factors related to the antimicrobial compound (B), factors related to the animal (C), and factors related to the farm (D). Each bar sums to 100%. The position on the X-axis indicates the skew of the distribution to the lesser or greater reported importance of each variable.


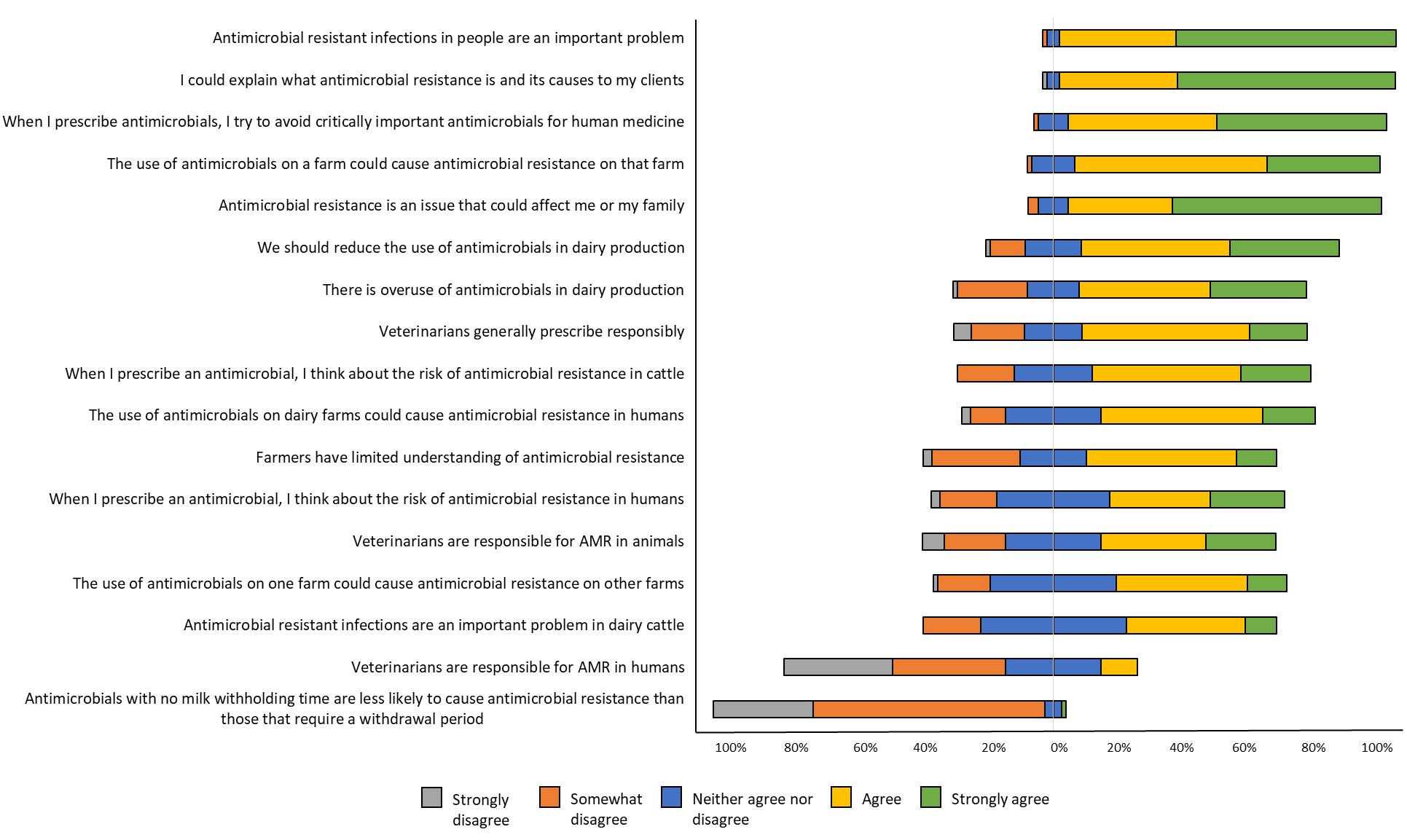


Supplementary Figure S2. Level of agreement of 82 dairy veterinarians with 18 statements related to awareness of antimicrobial resistance. Each bar sums to 100%. The position on the X-axis indicates the skew of the distribution to lesser or greater agreement with each statement.
